# Novel potential serological prostate cancer biomarkers using CT100+ cancer antigen microarray platform in a multi-cultural South African cohort

**DOI:** 10.18632/oncotarget.7359

**Published:** 2016-02-12

**Authors:** Henry A. Adeola, Muneerah Smith, Lisa Kaestner, Jonathan M. Blackburn, Luiz F. Zerbini

**Affiliations:** ^1^ International Centre for Genetic Engineering and Biotechnology, Cape Town, South Africa; ^2^ Faculty of Health Sciences, Division of Medical Biochemistry, Institute of Infectious Diseases & Molecular Medicine, University of Cape Town, Cape Town, South Africa; ^3^ Urology Department, Grootes Schuur Hospital, Cape Town, South Africa

**Keywords:** serum, prostate cancer, biomarkers, protein microarray, cancer-testis antigen

## Abstract

There is a growing need for high throughput diagnostic tools for early diagnosis and treatment monitoring of prostate cancer (PCa) in Africa. The role of cancer-testis antigens (CTAs) in PCa in men of African descent is poorly researched. Hence, we aimed to elucidate the role of 123 Tumour Associated Antigens (TAAs) using antigen microarray platform in blood samples (*N* = 67) from a South African PCa, Benign prostatic hyperplasia (BPH) and disease control (DC) cohort. Linear (fold-over-cutoff) and differential expression quantitation of autoantibody signal intensities were performed. Molecular signatures of candidate PCa antigen biomarkers were identified and analyzed for ethnic group variation. Potential cancer diagnostic and immunotherapeutic inferences were drawn. We identified a total of 41 potential diagnostic/therapeutic antigen biomarkers for PCa. By linear quantitation, four antigens, GAGE1, ROPN1, SPANXA1 and PRKCZ were found to have higher autoantibody titres in PCa serum as compared with BPH where MAGEB1 and PRKCZ were highly expressed. Also, p53 S15A and p53 S46A were found highly expressed in the disease control group. Statistical analysis by differential expression revealed twenty-four antigens as upregulated in PCa samples, while 11 were downregulated in comparison to BPH and DC (FDR = 0.01). FGFR2, COL6A1and CALM1 were verifiable biomarkers of PCa analysis using urinary shotgun proteomics. Functional pathway annotation of identified biomarkers revealed similar enrichment both at genomic and proteomic level and ethnic variations were observed. Cancer antigen arrays are emerging useful in potential diagnostic and immunotherapeutic antigen biomarker discovery.

## INTRODUCTION

Prostate cancer (PCa) is the leading cause of male cancer deaths in Africa [1], albeit little is known about the factors that determines its indolence or aggressiveness in this population. Factors suggested for aggressiveness of PCa in men of African descent includes; low educational levels, sociocultural beliefs, poverty and lack of healthcare infrastructure and manpower, *inter alia*. Greater research efforts are required to understand the molecular underpinnings of such disease disparity, albeit a higher level of testosterone [2, 3], variations in androgen receptor [3-5], dietary factors [6, 7], familial [8] and genetic mutations [8-11] have been suggested. Hence there is an urgent need for identification of minimally invasive, cost-effective and more reliable biomarkers for early detection, risk stratification, treatment efficacy and prognosis of disease.

Over the years, prostate cancer diagnosis has largely benefitted from the use of serological/blood-based biomarkers. For example, prostatic acid phosphatase (ACPP) and Prostate-specific antigen (PSA) have been beneficially used for diagnosis of prostate cancer. However, both ACPP and PSA fall short in their diagnostic ability for prostate cancer, particularly in the lower reference ranges (2-10ng/mL) where PSA is neither able to distinguish benign from cancerous prostate disease [12] nor distinguish indolent from aggressive phenotypes of the disease [13, 14]. Poor sensitivity for localized PCa was a major drawback for ACPP [15, 16], and its presence in extraprostatic tissue was another reason why its clinical use as a biomarker of PCa diminished [17, 18]. It is clear however that PSA measurement in combination with other molecular test can provide a more reliable clinical result. PSA derivatives such as velocity, doubling time, density, free-to-bound PSA ratio [19], kallikrein-marker panels, proPSA and Prostate Health Index (PHI) have been found to improve the diagnostic and prognostic value of PSA [19, 20]. Additionally, fusion of the ETS family member, ERG oncogene with androgen regulated TMPRSS2 gene has been suggested to be an important marker of aggressive PCa; albeit present in only about 50% of cases [19]. Circulating tumour cells (CTCs) have been used as blood-based predictor of distant PCa metastasis [21]; and urinary expression of prostate cancer antigen 3 (PCA3), sarcosine and TMPRSS2:ERG fusion are also emerging markers of PCa risk stratification [19, 21]. Evidently, a combination of good clinicopathologic assessment with a panel of reliable PCa biomarkers would be more beneficial. For instance, the use of multiparametric-MRI targeted prostate biopsy in combination with PHI and PSA isoforms, has been shown to improve PCa risk stratification as compared with urinary biomarkers or PSA alone [22].

Looking beyond the currently available methodologies for PCa biomarkers discovery, protein microarray technology is an emerging miniaturized high throughput option for multiplexed discovery of cancer biomarkers, molecular pathways and immunotherapeutic targets [23]. Considering that an important hallmark of natural immunity and cancer is the formation of autoantibodies [24], protein microarray technology in principle enhances the interrogation of an individual's humoral immune response in health and disease. Different forms of protein microarrays exist, including antibody arrays, microspot ELISA array, bead based arrays, reverse phase protein arrays and pathway arrays systems; arrays can be formulated on membranes, glass slides or beaded platforms [23]. This technology is potentially able to detect variations in antibody response to immunization and identify target antibodies in large cohorts of individuals [25]. Functional interrogation of a target subset of the proteome in a systems-oriented manner, economy of ligand or reaction quantity and consistency of the technical layout of array enables meaningful quantitation and comparison of protein microarray results across large datasets [26]. Several authors have demonstrated that the use of antibody-antigen microarray platforms is beneficial for biomarker discovery and early diagnosis of cancer [27-32].

Cancer/Testis antigens (CTAs) are a heterogeneous group of tumour associated antigens (TAAs) that are only found naturally in adult testicular and ovarian germ cells as well as placental trophoblast/embryonic membranes; and pathologically in cancers [33-36]. The restricted presence of these antigens in gonadal germline tissues and cancer makes them an attractive target for cancer diagnosis and immunotherapy. Over a hundred of CTAs have been described in scientific literature [35, 37] and their expression have been previously reported in melanomas, cancers involving the bladder, lungs, breast, prostate, kidneys, colon; as well as the lymphoreticular and hematologic systems [37]. The theranostic potential of CTA for urological malignancies was well explored in a review by Kulkarni *et al* [38].

Using seromic analysis of humoral adaptive response to cancer, potential CTA biomarkers and vaccine targets have been identified for non-small cell lung cancer [39], ovarian and pancreatic cancer [25]. Furthermore, CTAs have been previously reported as potential biomarkers of aggression [40, 41], disease progression [42], staging [43] and biochemical recurrence [44] of PCa. Some challenges of protein microarrays capture molecules include instability to pH alteration, variability in affinity and specificity for their target antigens, which is compounded by dynamic range issues of the plasma proteome. Furthermore non-active conformation of the arrayed protein can affect exposure of the desired epitope in a post-translationally modified protein [23]. In addition, most bioinformatics data analysis software for protein microarrays are adapted from genomic microarray computational workflows, which may not be directly amenable for individualized discrete immune response as found in antigen arrays [23, 25].

There is a paucity of literature on expression patterns and racial disparities of CTAs in heterogeneous African PCa cohorts. Hence, we describe herein a novel blood-based approach to potential PCa theranostic biomarker discovery using a protein microarray platform.

## RESULTS

### Potential biomarkers using linear analysis

**Figure 1:**
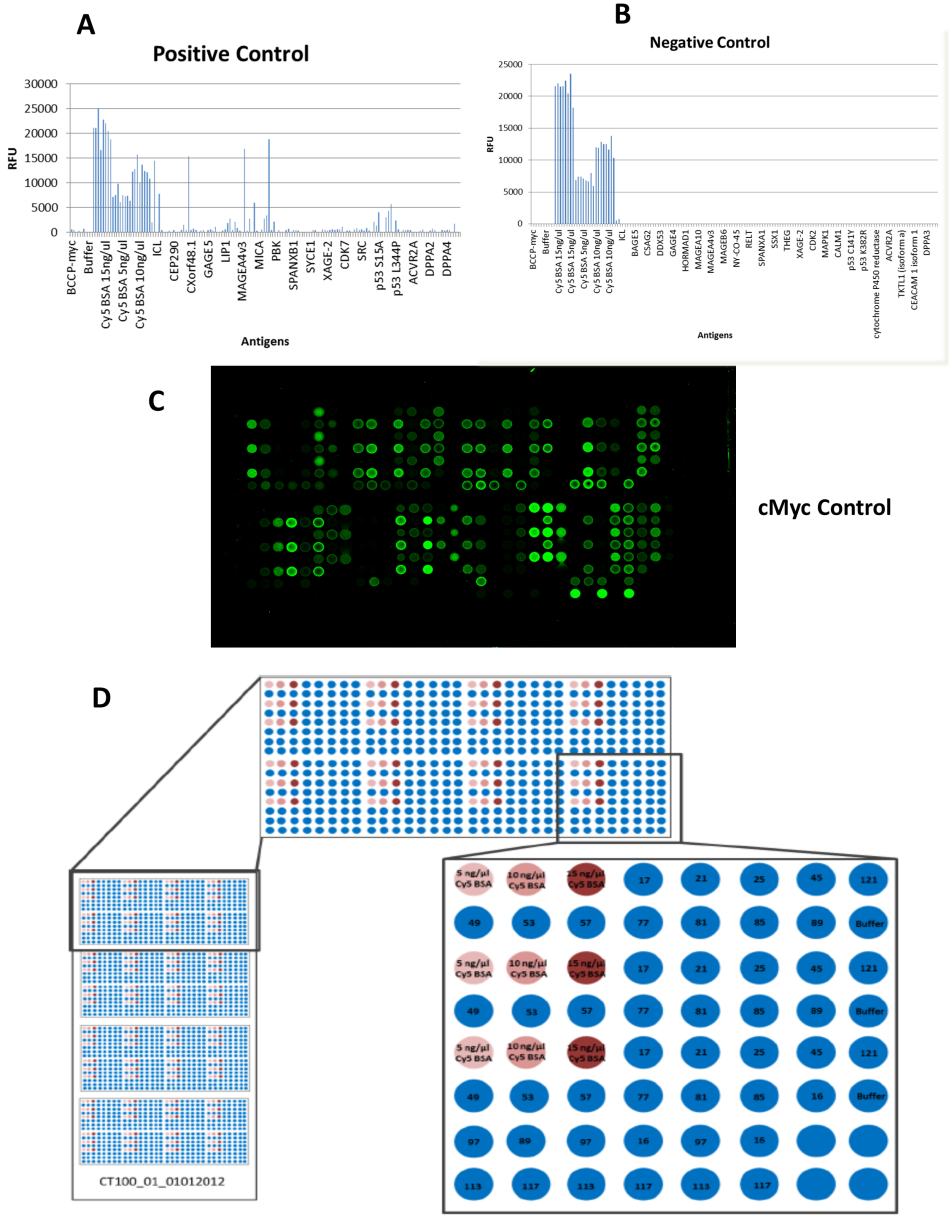
CT100+ cancer antigen microarray layout and quality control in experiment For quality control, **A.** pooled cancer sera derived from about 40 pooled multiple cancer sera showed positive antigenic signals while **B.** pooled non-cancer sera prepared from pooled serum samples derived from about 40 normal healthy individuals show negative antigenic signal. **C.** The anti-c-myc-Cy3 assay was used to confirm that individual antigens were successfully immobilised to the array during printing. **D.** Each Nexterion H slide is printed with a 4-plex subarray units and is further subdivided into 8 antigen printing units; capable of accommodating 64 antigen printing micro-spot per unit. Controls and antigens are printed in triplicates and antigen triplicates are printed on alternating and staggered spots. BSA-Cy5 control spots (pink and burgundy) are printed across the entire array for array normalization.

**Figure 2:**
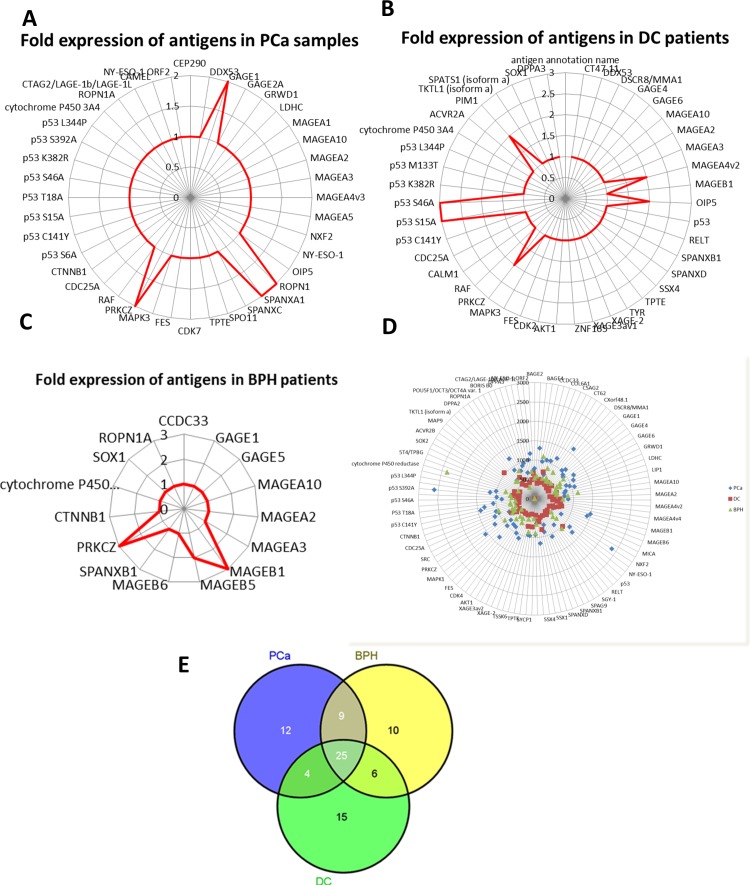
Linear fold-over-cutoff analysis **A.** Radar plot which helps assess performance of individual autoantibody response shows that GAGE1, ROPN1A, SPANXA1 and PRKCZ was highly expressed in PCa, while **B.** shows 2 mutant p53 (p53 S15A & p53 S46A) to be highly expressed in DC sera. **C.** Shows MAGEB1 and PRKCZ was highly detected in BPH sera. **D.** Radar plots showing difference in auto autoantibody response to CT100+ tumour associated antigens between PCa, BPH and DC groups. **E.** Venn diagram analysis revealed 12, 10 and 15 antigens unique to PCa, BPH and DC respectively.

Considering that most protein microarray analysis approaches are modified from gene microarray and given that statistical methods for protein microarray are currently evolving; absolute quantification of antigen for standardized comparison between different individuals can be challenging. We analysed a series of 67 patients' sera for autoantibody response to 123 antigens, composed primarily of a cocktail CTAs and a few other TAAs, and observed changes in autoantibody response in 41 of these TAAs using various analyses (Table 1). The positive control derived from about 40 pooled multiple cancer sera showed reactivity to many of the antigens on the array (Figure 1A), whereas the negative control prepared from pooled serum samples derived from about 40 normal healthy individuals showed no reactivity to antigens on the microarray (Figure 1B). The anti-c-myc-Cy3 assay was used to confirm that all the 123 individual antigens were successfully immobilised to the array during printing (Figure 1C). All 512 spots shown in Figure 1C are not of the same intensity because all 123 recombinant proteins are expressed to different degrees in the insect lysate and biologic processes like rate of degradation is not similar for all proteins on this array. They are linked to biotin and cMyc (positive control) to confirm their presence on the array, albeit all signals are not discernible by naked eyes. The signals of the apparently invisible spots are present and are read off by the ArrayPro Analyzer software and scored. For PCa samples, higher autoantibody titres were found to GAGE1, ROPN1, SPANXA1 and PRKCZ relative to other antigens (Figure 2A); while two mutant p53 antigen, p53 S15A and p53 S46A had the highest autoantibody titres in DC samples (Figure 2B). MAGEB1 and PRKCZ were found to have the highest autoantibody titres in BPH samples (Figure 2C). There was a general variation in autoantibody response to TAAs, observed between PCa and BPH and DC as shown (Figure 2D). These highly differentially expressed autoantibodies were confirmed by ranking the autoantibody responses according to their mean signal intensities and selecting the “top 20” intensities in each of the three categories for further analysis (Suppl. Table 1). By using this approach, SPANXA1 and p53 S46A were not found for PCa and DC groups respectively, possibly due to the fact that this method focuses on the signal strength and not necessarily presence or absence of autoantibody response. A third method of linear analysis which does to possess intensity components was performed using a three-way Venn diagram of the “top 50” autoantibody responses ranked by mean pixel intensity; which showed that 25 (50%) of the antigens were common to PCa, BPH and DC groups, 9 (18%) of the antigens were only common to PCa and BPH. There were 6 (12%) antigens only common to BPH and DC, whereas only 4 (8%) antigens were common to PCa and DC. A total of 12 (24%), 10 (20%) and 14 (28%) were found unique to PCa, BPH and DC respectively (Figure 2E). Interestingly, 6 (50%) of the 12 antigens found in PCa were found highly expressed by linear differential expression analyses. These 6 antigens were SPANXA1, p53 S392A, DPPA4, LDHC, FGFR2 and BORIS BO. Similarly CSAG2, which was one of the 10 antigens found unique to BPH was also found differentially expressed between PCa and BPH. Wild-type p53, DDX53, CT47.11 and p53 Q136X (a mutant p53) were found among the 14 antigens unique to DC (Suppl. Table 2). Top ranking 20 antigens in PCa, BPH and DC were identified and their mean intensities were documented (Suppl. Table 1). We selected the top 20 as the cutoff because we wanted mean antigen signals that were around 500 RFU and higher; which we observed in our analysis as indicating a truly high signal across many samples in a particular group.

**Table 1:** Characterization of 41 discovered potential serologic antigen biomarkers

S/N	Potential PCa antigen Biomarkers	Analyses	High/Low autoantibody titre in PCa	Ethnic Distribution
1.	DPPA4	Differential & Venn	High	MA
2.	CEACAM1 Isoform 1	Differential	High	CA
3.	NY-ESO-1	Differential & Top 20	High	MA
4.	FGFR2	Differential, Venn & Shotgun	High	CA
5.	RAF	Differential	High	MA
6.	ZNF165	Differential	High	
7.	TKTL1 (Isoform a)	Differential	High	IA
8.	MAPK3	Differential & Top 20	High	CA*
9.	CAMEL	Differential & Top 20	High	MA
10.	LDHC	Differential, Venn & Top 20	High	
11.	BORIS BO	Venn	High	
12.	SPANXA1	Linear & Venn	High	MA
13.	ROPN1A	Linear & Top 20	High	MA*
14.	p53 S392A	Venn	High	
15.	p53 L344P	Differential	High	IA
16.	p53 C141Y	Top 20	High	MA
17.	p53 K328R	Differential	High	MA
18.	p53 S15A	Linear & Top 20	Low	MA*
19.	p53 T18A	Top 20	High	MA*
20.	CDK2	Differential	High	IA*
21.	MAGEA11	Differential	High	CA
22.	FES	Differential & Top 20	High	
23.	OIP5	Differential & Top 20	High	MA*
24.	SSX2A	Differential	High	
25.	GAGE5	Differential	Low	IA
26.	MAGEB5	Differential & Top 20	Low	CA
27.	EGFR	Differential	Low	
28.	CCDC33	Differential	Low	CA
29.	CSAG2	Differential & Venn	Low	CA
30.	DDX53	Differential & Venn	Low	IA
31.	CT47.11	Differential & Venn	Low	IA
32.	p53	Differential & Venn	Low	
33.	p53 Q136X	Differential & Venn	Low	
34.	MAGEB6	Differential	Low	CA
35.	PBK	Differential	Low	IA*
36.	CAML1	Shotgun	High	IA*
37.	COL6A1	Shotgun	High	IA*
38.	GAGE1	Linear & Top 20	High	MA
39.	PRKCZ	Linear & Top 20	High	MA
40.	p53 S46A	Linear & Top 20	low	MA
41	MAGEB1	Linear & Top 20	Low	

Abbreviations: MA= Mixed Ancestry; IA= Indigenous African; CA= Caucasian African; S/N= Serial number; *=Significant

### Potential biomarkers by differential expression

**Figure 3:**
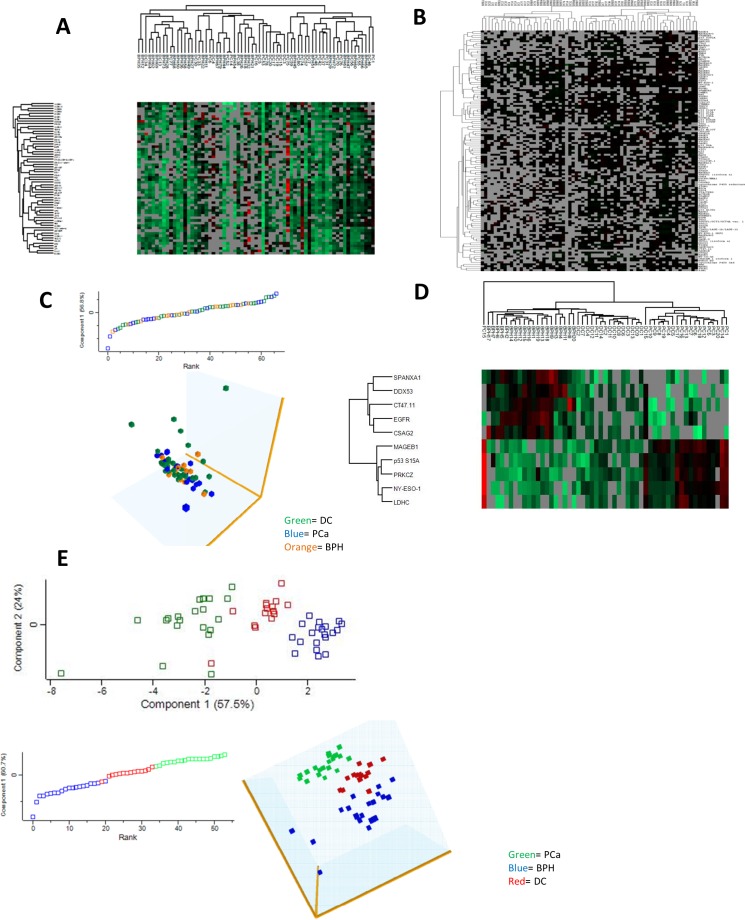
Differential expression analysis of potential antigen biomarkers of PCa Unsupervised hierarchical clustering showed no distinct class prediction both by **A.** Euclidian distance or **B.** *k*-mean correlation. Multivariate testing using principal component analysis showed similar trends, demonstrated by **C.** loading plot and 3-D plot. When Top 10 antigen for PCa by principal component were reanalyzed, **D.** distinct molecular signature were observed for PCa, BPH and DC by unsupervised hierarchical clustering as shown by heatmap. **E.** This distinct class clustering was also observed in multivariate principal component analysis both at 1-D, 2-D and 3-D plot.

We employed the Perseus Software (*version 1.4.0.20*), Cluster (*Version 3.0*) and TreeView (*Version 3.0*) for further bioinformatics analysis and visualization of the protein microarray data. Using an independent sample *t*-test with Bonferroni correction for multiple testing on the background corrected raw intensity data post-normalization; DPPA, CEACAM1 isoform1, NY-ESO-1, P53 L344P, GAGE5, MAGEB5, EGFR, CCDC33 and CSAG2 were differentially expressed (±2SD) between PCa and BPH (Suppl. Table 3A). We applied the same test to PCA and DC and identified 8 differentially expressed (±2SD) antigens including; P53 L344P, DPPA4, GAGE5, wild-type p53, RAF, ZNF, DDX53, and CT47.11 (Suppl. Table 3B). Considering that BPH is technically speaking, a form of disease control too, we further combined all the benign samples i.e. DC & BPH, and explored the differentially expressed antigens between benign and malignant samples. We found 17 TAAs with higher autoantibody titres in PCa in comparison to benign conditions, and 6 antigens were found with lower antibody titres accordingly (Suppl. Table 3C). A union of all identified potential biomarkers by linear, Top 20, Venn diagram, and differential expression analysis yielded a total of 41 potential antigen biomarker of PCa (Table 1). Initial unsupervised hierarchical clustering of all antigens using either Perseus or Cluster revealed moderate molecular signature overlap between PCa, DC or BPH (Figure 3A and 3B). This overlap was also observed in multivariate testing using principal component analysis (PCA), where individuals in distinct groups clustered haphazardly with other groups along the principal components both at 1-D and 3-D reconstruction (Figure 3C). However, on analysis with the top ranking 10 TAAs with the highest autoantibody titres and presence in the 20 PCa samples, distinct grouping patterns were identified for PCa, BPH and DC both using hierarchical clustering and PCA (Figure 3D and 3E). For hierarchical clustering, 17 (85%) of the 20 PCa patients clustered together, whilst two PCa patients (PC2 & PC11) clustered with DC and one PCa patient (PC15) clustered separately, albeit proximal to the BPH clusters. We used a traditional red-green colour scheme, where red stands for upregulation and green stand for down regulation in our hierarchical clustering. We re-evaluated the 2 PCa samples that clustered with DC and found that they had relatively lower PSA levels than other PCa cases. PC15 had a very high PSA level (315ng/mL), in spite of a moderate total Gleason score of 6. All BPH samples clustered distinctly together, while 13 (86.7%) of the 15 DC samples clustered together. Two of the DC samples (DC5 & DC15) clustered with PCa. On re-evaluation of these, DC5 was found to be of a younger age (34 years) and of African ethnicity (Black), however his PSA level was unavailable; DC15 was a 58 year old patient of Mixed-Ancestry ethnicity and a PSA level of 6.8ng/mL.

### Racial variations in PCa antigen biomarkers

**Figure 4:**
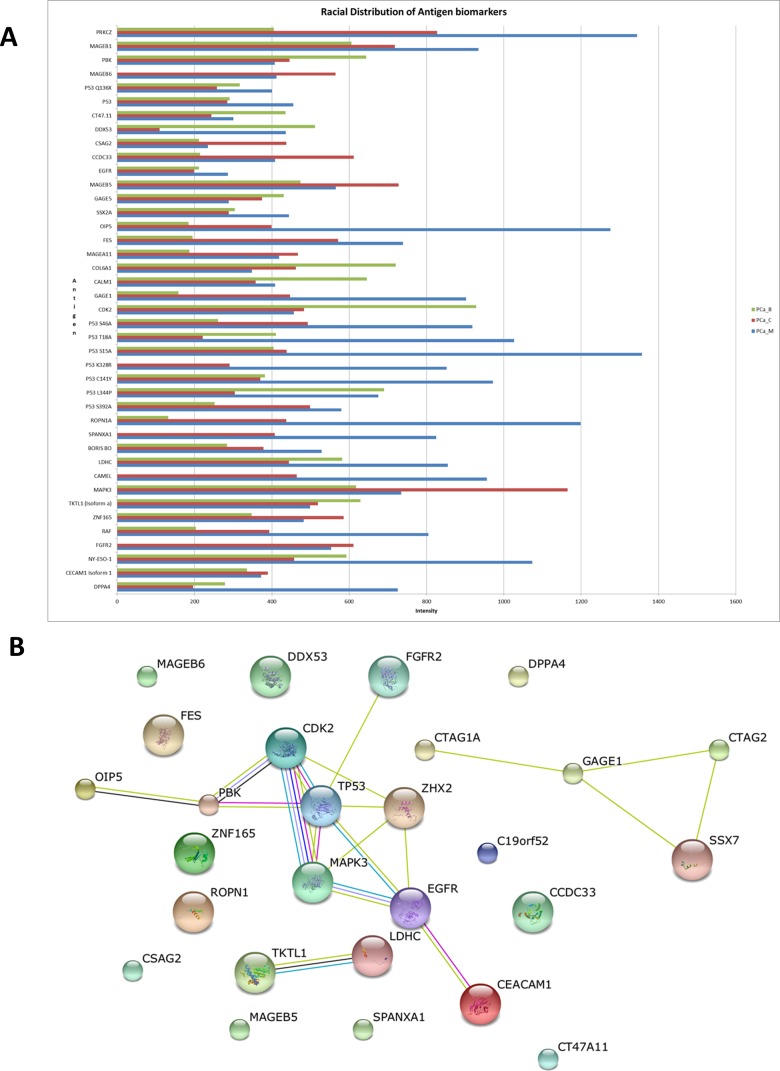
Functional pathway analysis and racial variation in potential antigen biomarkers **A.** Shows a bar graph of variation in mean antigen intensities between indigenous African (PCA_B), Caucasian African (PCA_C) and Mixed ancestry (PCA_M) prostate cancer sera. **B.** Pathway network annotation shows tumour-associated antigens (TAAs); CDK2, PBK, OIP5, MAPK3, EGFR, CEACAM1, and p53 having the highest connection.

We proceeded to determine if there was any variation in autoantibody response to TAAs between prostate cancer patients drawn from three major ethnicities of which our cohort was composed. There were 3 samples from Indigenous African (Black), 6 from Caucasian African (White) and patients, and 11 from Mixed-Ancestry (Coloured) PCa patients. All 41 potential antigens biomarkers were examined in our PCa patient cohort. We observed variation in autoantibody response to TAAs between the Caucasian African (PCa_C), Indigenous Africans (PCa_B) and Mixed Ancestry (PCa_M) prostate cancer patients (Figure 4A). The mixed ancestry population has the highest autoantibody expression, which included DPPA4, NY-ESO-1, RAF, CAMEL, SPANXA1, ROPN1A, GAGE1, OIP5, PRKCZ and a subset of mutant p53 antigen including p53 S15A, p53 T18A, p53 S46A, p53 K328R and p53 C141Y. Among these 14 antigen, OIP5, ROPN1A, p53 S15A and p53 T18A had the highest autoantibody response in PCa_M as compared with PCa_B or PCa_C. There were 8 highly expressed autoantibodies for PCa_C in comparison to PCa_B or PCa_M, which included MAGEB6, CSAG2, CCDC33, MAGEB5, MAGEA11, MAKP3, FGFR2 and CEACAM1 Isoform 1. MAPK3 was found to be significantly more expressed in PCa_C as compared with the others. Nine antigens were found to have a high autoantibody titre in PCa_B, which included PBK, CT47.11, DDX53, GAGE5, COL6A1, CALM1, CDK2, p53 L344P and TKTL Isoform a. Among these, PBK, COL6A1, CALM1 and CDK2 were found with the highest autoantibody titre in PCa_B.

### Functional characterization of potential PCa antigen biomarkers

The potential of these differentially expressed CTAs and TAAs to be involved in common or multiple biologic pathways was investigated to further understand and explore functional pathways in PCa pathogenesis. Gene names for potential antigen biomarkers for PCa were uploaded into GeneMANIA (http://www.genemania.org/) and bioinformatics enrichment analysis for the GO terms cellular component, molecular function and biological process was automatically carried out. Functionally clustering the “biological process” as the major Gene Ontology (GO) term for enrichment in the differentially expressed antigens revealed that they act *via* similar signal transduction pathways involving: Fc receptor, fibroblast growth receptor, epidermal growth factor receptor, ERBB, neurotrophin, ERK1/ERK2 signaling pathways. These 6 signaling pathways were found common to the lists of differentially expressed antigen between PCa and DC or between PCa and all controls (DC and BPH) (Suppl. Table 4A & 4B). When the antigens unique to PCa (judged by Venn diagram analysis) were queried, cellular processes involved in reproduction were the major pathways involved (Suppl. Table 4C). Cellular process involved in reproduction was also one of the top 25 pathways common to “PCa *vs* all controls”. In order to determine whether this was the true picture at the protein expression level, we queried the same complement of genes using STRING (*version 9.1*) functional protein association network software. Using this approach, we found that similar molecular signaling pathways were involved both at the gene and protein expression level. (Suppl. Table 4D). Network generated from STRING demonstrated that TP53, CDK2, MAPK3, PBK, and CEACAM1 were strongly linked TAAs by neighborhood evidence (Figure 4B). Even though some CTAs were not shown to be physically interacting with this TAA network, the possibility of their co-expression or predicted linkage is plausible. In addition, CTAG1A, GAGE1, CTAG2 and SSX7 were found to be linked by neighborhood evidence. We also observed co-occurrence, neighborhood and experimental evidence for TKTL1 and LDHC.

### Shotgun proteomics data verification

We sought to assess the presence of CTA and TAA expression in urine of PCa patients in our cohort. We screened our previously acquired urinary shotgun proteomics data [45] for all antigens on our CT100+ array. Out of the 123 antigens on our array, we found only 11 (8.9%) antigens in the urine of our PCa patient cohort. These included FGFR2, MAPK1, COL6A1, SOX1, CALM1, SRC, LIP1, CEACAM1 isoform 1, RELT, EGFR and ITGB1 (Suppl. Table 5). Further focusing on antigens without splice variants in the shotgun data, only 5 (4.5%) antigens namely COL6A1, RELT, CALM1, FGFR2 and ITGB1 were found. Preliminary classification of the identified antigens based on their differential expression both in the cancer antigen array and shotgun proteomics experiment showed that of these 5 antigens, only COL6A1, FGFR2 and CALM1 demonstrated promise as biomarkers of PCa by both approaches.

## DISCUSSION

It is well known that cancer interferes with both the adaptive and innate immunity. In this study, we used a novel cancer antigen microarray platform to explore serum autoantibodies to TAAs as potential biomarkers and immunotherapeutic target for PCa in our South Africa cohort, with a specific focus on CTAs. Owing to wide variations in the size and abundance of proteins present in serum [46], it is important to evaluate the dynamic range and sensitivity of the microarray used in this study. Earlier protein microarray platforms were limited by narrow dynamic range and low sensitivity for analytes of interest [47, 48]. However, significant improvement in dynamic range of newer protein microarrays and their sensitivity in the femtomolar range have increased their relevance [48]. Using a two-step linearity and dynamic range assay as described in our previous work [36], we have demonstrated linear response in dynamic range of over 3 orders of magnitude. In addition, using a noise threshold of 2 standard deviation (of the background) in this previous study, we observed a detection limit of ca. 1:1,000,000 serum dilution, which corresponded to an autoantibody titer of about 190pg/mL [36].

Autoantibodies are more stable in serum than polynucleotides or proteins which may be quickly degraded soon after their release by tumour cells [49]. Autoantibodies are capable of remaining in the blood even long after the removal of antigenic stimulus [50], and they represents a better measurable biosensor that can be correlated with different disease and health states; albeit we recognize the fact that autoantibody response between individuals is dynamic. Hence, measurement of autoantibody response to TAAs is a better approach than the direct measurement of serum autoantigen. Due to the detection of a few of the arrayed TAAs in our previous shotgun urinary proteomics data, we assumed that it is plausible that an increased autoantibody titre may be indirectly associated with increased expression of the autoantigen.

Although the renal glomerular filtration barrier was anecdotally suggested to prevent the passage of macromolecules such as proteins due to a smaller pore size in comparison to serum protein size [51], there is overwhelming literature evidence for the presence of pathogen-specific antigens in urine [52-55]. Suggested barriers to protein filtration include pore size, charge selectivity, shape of protein and potential for downstream tubular reabsorption [56, 57]. Even though, it was reported that the human nephron size selectivity is about 50KDa [58]; the molecular weight of the overlapping TAAs identified in urine in this study ranged from 39-140KDa. This suggests the possibility of an alternative mechanism such as shape/structure might explain their presence in urine. Another important consideration is that we may be detecting proteolytic fragments or “degradome” of full length proteins in our shotgun urinary proteomics data. Emerging evidence suggests that low molecular mass proteins/antigens are able to pass through this filtration barrier and are concentrated in the renal proximal tubule [58]; and then sequestered in the renal lymph nodes and dendritic cells. Such antigens stimulate PDL1-mediated apoptosis of antigen presenting T-cell. Hence there is physiologic hyporesponsiveness, inactivation or clonal deletion of cytotoxic T-cells against circulating innocuous proteins/antigen [58-60]. It is therefore plausible that that the low overlap between serum and urinary antigen stems from a combination of lability of serum antigens, renal filtration barrier, dendritic cells/renal lymph node sequestration, *inter alia*. It therefore seems reasonable that urinary cancer antigen testing may become more routinely used in clinics for cancer diagnosis in the future.

Many antigens on our platform have been specifically documented to be dysregulated in PCa, albeit all are cancer-associated. For instance, promoter methylation of GAGE1 has been reported in PCa cell lines [61], while ROPN1 has been found to be expressed in a subset of acute myeloid leukemia (AML) patients [62]. SPANXA1 a member of the SPANX-A/D cluster of SPANX gene located on Xq27 [63] has been found highly expressed in cancer as well. Recently, a splice variant of PRKCZ has also been shown to be an emerging biomarker of malignant prostatic epithelium as well as PCa cell lines [64].

It is unclear why immunoreactivity of BPH sera to PRKCZ was high in our cohort; possibly indicating a malignant biotransformation potential of BPH. In addition, MAGEB1, which also had a high BPH sera immunoreactivity in our study was previously found to exhibit low immunoreactivity to PCa after administration of Lenalidomide (a thalidomide analogue) to PCa patients in a randomized phase II trial [65]. Generally p53 missence mutations were found more frequently in PCa and BPH sera, while wild-type p53 was found more commonly in DC. Two missence mutations p53 S15A and p53 S46A found in DC have been previously reported to be responsible for inactivation of constitutive phosphorylation of p53 [66] and dysregulation of apoptotic target genes, cell cycle, senescence as well as suppression of ERK activation [67, 68], respectively. Phosphorylation of p53 S46 appears to be important for maintaining genomic stability of cells after DNA damage and its high autoantibody response in our DC sera may indicate that a follow-up should be directed at patients who, despite presenting with symptom of prostate disease, screened negative histopathologically. There is evidence that anti-p53 (auto) antibodies isolated from cancer patients are sometimes unable to recognize wild-type p53; and most commercial antibodies and vaccines are directed to the p53 terminal domain epitopes which is similar in both wild-type and mutant p53 rather than the “core” DNA-binding domain where most of the mutations occur [69]. These observations support the idea that differential signals to p53 variants on our array are real We found considerable overlap between potential antigen biomarkers of PCa identified by different analyses, notably BORIS BO which was unique to PCa in our 3-way Venn diagram analysis has been reported recently to correlate at mRNA level with prostate cancer aggression and is potentially able to activate the androgen receptor genes [43].

Class comparison of differentially expressed antigen in our study showed that DPPA4, a nuclear chromatin associated embryonic stem cell protein was more upregulated in PCa sera compared to BPH and DC. Expression of DPPA4 has been reported in PCa cell lines but its role in somatic cancer is largely unknown [70]. Higher titer of NY-ESO-1 antigen in PCa serum in comparison to BPH and DC demonstrates that this antigen which is known to generate autoantibody response in many human cancers, could be a part of diagnostic antigen biomarker panel for PCa. We found the autoantibody response to CSAG2 antigen to be lower in our PCa sera in comparison to BPH, albeit previous reports did not find any difference in CSAG2 gene expression between PCa and normal prostate tissue [44]. GAGE5 antigen which had a low expression in our PCa sera compared to BPH and DC was reported not to be significantly expressed by SKOV-3 human ovarian cancer cell lines [71]. Using unsupervised hierarchical clustering and testing of multivariate predictors using principal component analysis of the top 10 most representative PCa antigen predictor showed clear-cut molecular signature between PCa, BPH and DC.

Variation in antigen expression was observed here between indigenous African, Mixed Ancestry and Caucasian PCa patients in our cohort. Notably, ROPN1A, OIP5 and two mutant p53 antigens (S15A & T18A) were highly expressed in PCa patients of Mixed Ancestry origin. Mutation of Thr18 to alanine in p53 using site-directed mutagenesis has been reported to result in the reduction of thioredoxin reductase expression, DNA damage, increased expression of p21, and increased production of reactive oxygen species [72]. In a report by Lee *et al* [73], OIP5 has been described as a targetable CTA for colon and gastric cancer using cell lines and cancer tissues. MAPK3 was found to be significantly highly expressed in Caucasian patients in our cohort in comparison to Indigenous Africans or Mixed Ancestry PCa patient. We observed that CSAG2, even though previously reported to be highly expressed in prostate cancer [40], was generally higher in our BPH as compared with PCa sera. However, focusing on our PCa data alone we observed that CSAG2 was higher in the Caucasian sera, as compared with other racial groups. This difference is possibly due to the population from which these samples were drawn and the fact that formalin fixed paraffin embedded (FFPE) sections were used in previous studies [40]. In addition, our cohort may not be large enough to conclude that CSAG2 is lower in African PCa patients as compared to their Caucasian counterparts. Hence, this observation warrants further studies using samples drawn from African PCa patients' populations. We also found COL6A1, CALM1, PBK and CDK2 to be highly expressed in Indigenous African PCa patients in comparison to Caucasian and Mixed Ancestry PCa patients in our cohort. PBK and CSAG were found to correlate significantly with histopathologic progression using Gleason's score in the same study [40]. Taking advantage of racial variation in TAA antigen expression may be a plausible approach to PCa theranostics in the foreseeable future.

It was evident from pathway analysis that the strong link between CDK2, MAPK3, EGFR, CEACAM1, PBK, OIP5, FGFR and TP53 seem to play crucial roles in the pathogenetic mechanism of PCa development. In addition, other networks such as CTAG2, GAGE1, SSX7 and CTAG1A; as well as TKTL1 and LDHC need further study *vis-à-vis* PCa pathogenesis. It is also noteworthy that some of the potential serological TAA biomarkers of PCa were also identified in our previous urinary shotgun discovery proteomics data [45]. This observation makes sense because the majority of our potential urinary biomarkers were predicted membrane and intercellular matrix/space proteins and the process of cancer development involves disruption of cell-cell contact and discohesiveness of the normal prostate epithelial architecture.

Even though ELISA has been widely known as the “gold standard” for confirmatory assessment of antibody interaction, our platform has been demonstrated to be at least as sensitive as ELISA and more capable of multiplexing. In addition, the specificity of the arrayed antigens has been confirmed by western blot assays [36]. An interesting approach to confirmation would be to develop a multiplexed mini-array of fully validated biomarker subsets for in-vitro clinical immunodiagnostics. A panel of antigens on the full array to which autoantibody response is found can be miniaturized for point-of-care (POC) diagnostics. Such mini arrays have been previously used for confirmatory identification of differential autoantibody response signature for Hepatocellular [74], Ovarian [75], Breast [76], and Colon [77] cancer. The cohort of differential autoantibody response signature discovered for prostate cancer in the current study could serve as a basis for its immunodiagnostic mini-array fabrication.

In this study, we explored potential serologic diagnostic biomarkers and immunotherapeutic targets for PCa in sub-Saharan Africa (SSA), a continent majorly constituted of low and middle income countries (LMIC), and where infection plays a major role in cancer development [78, 79]. It is envisaged that cancer vaccination would play an important role in reduction of the cancer burden in SSA. Emerging cancer immunotherapy techniques such as checkpoint blocking antibodies [80] and genetically engineered T-cells [81] are beginning to take center stage, albeit great attention needs to be paid to possible side effects. There are various vaccination-based therapies currently in the pipeline for different stages of PCa [82], but several bottlenecks need to be addressed to bring these to routine clinical application [83]. Our work serves as a foundation for further immunological PCa theranostic research in Africa and development of point-of-care (POC) tools for timely diagnosis and treatment monitoring of PCa, as well as for patient stratification prior to therapeutic vaccination.

## MATERIALS AND METHODS

### Patient cohort information and sample collection

Full ethical approval (*Approval # HREC454/2012*) for study was obtained from the Human Research Ethics Committee of the Faculty of Health Sciences, University of Cape Town, South Africa. Blood samples (*N* = 67) were collected spanning a 2 year period from patients diagnosed with prostate cancer (PCa) (*n* = 20), benign prostatic hyperplasia (BPH) (*n* = 32), and symptomatic individuals who had other uropathies or were histopathologically screened as negative for BPH or malignancy (DC) (*n* = 15). All participants attended the urology clinic of Grootes Schuur Hospital, Cape Town, as well as two subsidiary centres: New Sommerset Hospital and the Eerste Rivier Hospital. Comprehensive informed consent was obtained from all participants with strict adherence to the principles of the Declaration of Helsinki (DoH) 2008. Demographic and Clinicopathologic data of the PCa patients were documented. (Table 2) After counselling, about 2-3mL of blood was obtained with Vacuette^®^ K2EDTA venous blood collection set (Greiner Bio-One, North Carolina, USA) from participants and stored on ice at the clinic and subsequently transported rapidly to the laboratory for processing. In the laboratory, samples were centrifuged at 1,500 × g for 15 minutes to separate the plasma from cell fraction. The supernatant plasma fraction was carefully removed and stored in 2ml cryotubes at −80°C until experiments were performed.

**Table 2:** Clinicopathologic information on all 67 patients used for the study

Code	Diagnosis	Age	PSA level	Race Group	Gleeson Score
PC1	CANCER	61	11.6	MA	6
PC2	CANCER	71	5.1	MA	7
PC3`	CANCER	61	100	B	9
PC4	CANCER	74	31	MA	7
PC5	CANCER	61	16.4	MA	6
PC6	CANCER	67	350	MA	9
PC7	CANCER	80	39.4	B	7
PC8	CANCER	68	1091	MA	9
PC9	CANCER	69	5.9	W	7
PC10	CANCER	64	9.1	W	6
PC11	CANCER	64	1.1	W	6
PC12	CANCER	76	195	W	10
PC13	CANCER	74	1.5	MA	6
PC14	CANCER	66	34	MA	9
PC15	CANCER	76	315	MA	6
PC16	CANCER	62	17.05	MA	7
PC17	CANCER	63	24.05	MA	7
PC18	CANCER	71	2.9	W	9
PC19	CANCER	74	184	W	10
PC20	CANCER	77	738	B	8
BPH1	BENIGN	72	3.6	MA	NA
BPH2	BENIGN	64	2.8	MA	NA
BPH3	BENIGN	70	5.4	W	NA
BPH4	BENIGN	61	2.9	MA	NA
BPH5	BENIGN	75	3.7	MA	NA
BPH6	BENIGN	58	19.5	W	NA
BPH7	BENIGN	70	48.4	MA	NA
BPH8	BENIGN	68	5.7	MA	NA
BPH9	BENIGN	69	7.1	MA	NA
BPH10	BENIGN	56	5.03	MA	NA
BPH11	BENIGN	53	1.24	W	NA
BPH12	BENIGN	63	4.7	W	NA
BPH13	BENIGN	86	10.6	B	NA
BPH14	BENIGN	56	9.6	MA	NA
BPH15	BENIGN	75	7.3	B	NA
BPH16	BENIGN	61	4.5	B	NA
BPH17	BENIGN	81	25.8	B	NA
BPH18	BENIGN	64	6.6	B	NA
BPH19	BENIGN	74	2.6	B	NA
BPH20	BENIGN	57	3.64	MA	NA
BPH21	BENIGN	69	3.3	MA	NA
BPH22	BENIGN	70	1.2	W	NA
BPH23	BENIGN	75	0.83	W	NA
BPH24	BENIGN	65	27	B	NA
BPH25	BENIGN	68	5.8	MA	NA
BPH26	BENIGN	62	1.4	W	NA
BPH27	BENIGN	78	0.1	B	NA
BPH28	BENIGN	66	3.6	W	NA
BPH29	BENIGN	68	0.75	B	NA
BPH30	BENIGN	70	9.1	MA	NA
BPH31	BENIGN	77	37.1	MA	NA
BPH32	BENIGN	67	6.43	MA	NA
DC1	NFM	44	0.7	MA	NA
DC2	NFM	74	7.8	MA	NA
DC3	NFM	62	3.7	MA	NA
DC4	NFM	52	NA	MA	NA
DC5	NFM	34	NA	B	NA
DC6	NFM	63	6.3	MA	NA
DC7	NFM	67	5.9	MA	NA
DC8	NFM	65	3.4	MA	NA
DC9	NFM	59	18.9	MA	NA
DC10	NFM	69	0.4	MA	NA
DC11	NFM	61	8	MA	NA
DC12	NFM	76	7	B	NA
DC13	NFM	56	0.9	MA	NA
DC14	NFM	35	NA	B	NA
DC15	NFM	58	6.5	MA	NA

Abbreviations: PSA= Prostate-specific antigen; MA= Mixed Ancestry; W= White; B= Black; NFM= Negative for malignancy

### In-House derivatisation of nexterion H-slide microarray

A black barcoded, nitrocellulose hydrogel coated Nexterion^®^ Slide H (SCHOTT GmBH, Jena, Germany) with dimensions 75.6mm × 25mm × 1.0mm and thickness 1.0±0.5mm was used was used for cancer antigen printing due to its suitability for covalent immobilization of capture molecules. These slides which were initially stored at −20°C were thawed for 1 hour at room temperature. Each slide was immersed in 5 ml of 1 mg/mL streptavidin solution (in 150 mM Na2HPO4 buffer, pH 8.5) for 1 hour at room temperature. The slides were then removed from the streptavidin solution and washed for 1 hour in 10 ml 150 mM Na2HPO4 buffer (pH 8.5) containing 50 mM ethanolamine (blocking reagent). Following this, each slide was washed thrice for 5 minutes in 10 ml washing buffer (containing 0.05% Tween20^®^) and then washed for 10 minutes in 10 ml of water. Individual slides were dried through centrifugation at 1000 ×g for 5 minutes at room temperature and then transferred to slide boxes, sealed in foil bags and stored at −20°C until use.

### Quality control of streptavidin-derivatised slides

A quality control (QC) test was performed to ensure a homogenous spread of streptavidin on the Nexterion H-slides. For the QC test, the last slide per batch derivatised was incubated with 10 μg/ml Cy5-biotinylated BSA (in PBS), washed in washing buffer and scanned using the Tecan LS Reloaded^TM^ microarray scanner (Tecan Group Ltd, Männedorf, Germany). Slides were used for assays if the CV was ≤ 5% across the slide surface.

### CT100+ antigen microarray fabrication

Antigen-containing crude insect lysates were printed on the streptavidin-coated Nexterion H-slides using the Genetix QArray2 robotic arrayer (Genetix Ltd., New Milton, UK), a high throughput microarray printer. In general, heterologous expression using *Escherichia coli* has been plagued with expression problems like protein solubility, absence of posttranslational modifications (PTMs), and defects in folding [84]. Hence, we have used *Spodoptera frugiperda* SF21 insect cells to express our human antigens of interest because it presents a simple eukaryotic-like expression system that better preserves native protein folding and PTMs [26]. Full details about our optimized insect expression system and lysate preparation are well documented in our previous works [26, 36, 84]. The physical basis of ligand binding assay system is made up of spatially-defined array of immobilised, purified CT antigens printed on a surface which enables cognate autoantigens capture in serum sample. Anti-CT antigen expression is then detected through anti-human IgG, fluorescently labelled and captured on the microarray surface. The positive controls included biotinylated human IgG and biotinylated human serum, while the negative control consists of a crude cell lysate containing the BCCP-tag alone with no recombinant fusion partner. All controls were prepared in lysis buffer (25 mM HEPES pH 7.5, 20 % Glycerol, 50 mM KCl, 0.1 % Triton X-100, 0.1 % BSA, 1 mM DTT and 50 μM biotin) supplemented with 20 % sucrose. In addition, each subarray on the microarray contained triplicate biotinylated Cy5-BSA (at 5, 10 and 15 ng/ml) spots, used for slide orientation and signal normalisation methods.

### QC in CT 100+ antigen microarray fabrication

Before the printing procedure, each slide was carefully inspected. Here, it was confirmed that slides were free of any forms of contamination e.g. dust particles, fingerprints, etc. The Qarray2 arrayer is equipped with 300 μM solid pins to print replica CT100 arrays in a 4-plex format. Lysates were mixed with an equal volume of 40 % sucrose and spotted in triplicate in each array. The microarray printing settings are described as follows: arraying pattern = 8 × 8 (16 pins, 4 fields); the row/column pitch = 562 micron; maximum stamps per ink = 1; number of stamps per spot = 1; stamp time = 0; inking time = 500 ms; print depth adjustment = 150 microns; number of touch-offs = 0; water washes = 60s (dry time = 0 ms); ethanol wash = 10s (dry time = 10s). After the printing procedure, each slide was washed for 30 minutes with 50 ml pre-chilled blocking solution (25 mM HEPES pH 7.5, 20 % Glycerol, 50 mM KCl, 0.1 % Triton X-100, 0.1 % BSA, 1 mM DTT and 50 μM biotin), washed 3 × 5 minutes with PBST, and a 1 × 5 minute rinse. The slides were then stored at −20°C in storage buffer (Blocking buffer with 50 % glycerol) until assays were performed.

### CT100+ assay

A total of 70 samples were assayed, of which 3 were used for quality control purposes. These QCs included positive control serum, negative control serum and a mouse-anti-c-Myc-Cy3 antibody. The positive control showed reactivity to a number of antigens on the array, whereas the negative control showed no reactivity to antigens on the microarray. The anti-c-myc-Cy3 assay was used to confirm that all the 123 individual antigens (listed in Suppl. Figure 1) were successfully immobilised to the array during printing (Layout demonstrated in Figure 1D). The entire printed array is a 4-plex, and each 1-plex of the entire array is made up of 8 antigen printing subarray unit containing 64 spots each. Hence, each 1-plex is made up of 512 (64 × 8) spots. As shown in Figure 1D, BSA-Cy5 control spots (pink and burgundy) are printed across the entire array for array normalization. For the assay, the CT100+ microarray was washed with lysis buffer (25 mM HEPES pH 7.5, 20 % Glycerol, 50 mM KCl, 0.1 % Triton X-100, 0.1 % BSA, 1 mM DTT and 50 μM biotin) for 3 × 5 minutes at room temperature. The slides were then dried through centrifugation at 240 RPM for 2 minutes. Thereafter, each slide was placed in the Tecan HS4800 Pro automated hybridiation station (Tecan Group Ltd, Männedorf, Germany) as a means to isolate each array. In the hybridisation station, each microarray was incubated with sera (diluted 1 μl sera in 800 μl PBST) for 1 hour at 23°C. Once the incubation period was concluded, each microarray was washed for 3 × 5 minutes with PBST. Each individual microarray was incubated with detection antibody (Cy5-goat anti-human IgG diluted 1:100 in PBST) for 1 hour at 23°C. The slides were then washed for 4 × 1 minutes with PBST followed by a 30 second water rinse. Individual arrays on each slide were scanned with 10 um resolution using the Tecan LS Reloaded microarray scanner, and fluorescence was detected. Each image was then saved as a 16-bit TIF file (Suppl. Figure 2). All samples that showed spot merging of Cy5-biotin-BSA channels were re-assayed.

### TIFF images and data extraction

The resulting TIFF files from the scanning procedure were subjected to visual inspection analysis and quality control. Here, we confirmed that the BSA spots at the three different concentrations (5, 10 and 15 μg/ml) were visible within each subarray of the microarray. Human IgG (detected by fluorescently labelled secondary antibody) and human anti-IgG (detected only when plasma or serum is added to the slide) were used as positive controls to assess image signal intensity. A visual inspection was performed for each microarray and we excluded any arrays which showed high background, excessive speckling, presence of interfering dust particles, and evidence of protein spot coalescing. If any of the arrays displayed the aforementioned properties, the sera were re-assayed in fresh slides until the anomaly is corrected. The data was extracted using the Array-Pro 4.5 (Media Cybernetics, Inc., Maryland, USA). A grid, containing the identity of each antigen and control, was aligned such that it encircled each antigen and control on the TIFF image. The raw intensity of the individual spots was measured as a mean pixel within the spot border, with a maximum of 200 pixel intensity set as the threshold. The background for each spot was measured as the intensity of the area adjacent to the circumference of each spot (i.e. local background). The mean net intensity of each spot was calculated as the difference of the raw mean intensity and its local background. Once the data was extrapolated by ArrayPro Analyzer Software, it was filtered and normalized using an in-house developed software (CT100+ programme) with additional data analysis.

### CT100+ programme

An important focus of the CT100+ programme was to address the bottlenecks of protein microarray data normalisation, class comparison and qualitative clustering of data. The current programme developed in-house [84] aims to correct various parameters as discussed in the following subsections.

### Spot related QC

Technical variations in triplicate signals from spot to spot should be identical. Multiple spots may bleed into each other due to closeness of the spots as well as using the wrong buffer at a particular spot. Another issue may involve the spotting pin getting stuck during print runs or due to wrong calibration and artefact formation due to poor cleaning of print-heads between runs. Individual spot signals are expected to have pixel homogeneity across spot surface. Pin height errors, improper handling and storage condition may result in “doughnut effect”, high intensity spots, printed spot evaporation and uneven spot intensity. We evaluated homogeneity by estimating the coefficient of variation (CV) and percentage mean intensity within a spot; and any spot that falls short of the expected quality is repeated.

### Background signal correction

Variation in background signal across array spots should be minimal. Improper handling, dust particles, poor storage and presence of artefacts can result in high background signal at a print spot. This reduction in “signal to noise ratio” (SNR) makes it difficult to identify genuine signals (net spot intensity) because there is no distinct difference in the intensity of the high background noise and the actual foreground signal intensity. For our QC, only SNR of nothing less than 2 is acceptable for further statistical evaluation. Spots across the array should not contain saturated pixels as this can affect the scanner reading. When there is a faulty array the whole process of sample preparation, microarray printing and CT100+ assay is repeated. If high signals still persist after repeat assays, automatic gain control (AGC) is used to ensure that the signal lies within the reference range of 200-65,550 RFU. If AGC signal is still too strong after this, the measurement may go into the non-linear range of the platform; hence the antigen is flagged automatically. This is a preliminary quality control measure and further normalization is subsequently carried out by the array analysis software programme. The same scanner was used for all the slides throughout this study.

### Cancer antigen array data filtering

Further QC was performed to filter out noise and array defects before bioinformatics evaluation. The quality of data is improved by filtering because poor quality and disputable spot or arrays can be easily detected. Spots with saturation levels beyond our saturation cutoff were excluded from analysis as well as triplicate spots intensity with high CV. When this happens, the spot with high intensity is excluded and analysis is carried out on the remaining two spots (S1 and S2). The variability is now defined by the equation (|S 1 − S 2|)/(S 1 + S 2).

### Intensity normalization workflow

Prior to data analysis, normalization was carried out using a customized composite normalization method for antigen arrays. This method is robust enough to accommodate a relatively smaller number of positive controls to inter-array and inter-spot variation. The assumption we made here was that there is equal distribution of intensity in all positive controls across array. Based on this proviso, a combination of quantile normalization and total intensity normalization was performed to eliminate systematic bias on the array. In the quantile normalization workflow, the Cy5-labelled positive controls are assumed to share similar distribution across arrays. This distribution is used to define baseline “house-keeping” intensities across arrays. The data is then reorganized to accommodate outlier spots in the positive control dataset. In the total intensity normalization workflow, summation of all positive controls on each array is expected to be constant. If a “house-keeping” positive control spot intensity is considered to be an outlier, this normalization method ensures that the same spot is regarded as such across all arrays.

### Linear and differential statistical analysis

After the preprocessing statistical step using the CT100+ programme, comparative spot intensities analyses between the PCa, BPH and DC were performed and a set of top ranking antigen in each group with linear fold over cut-off differences in relation to the interquartile values per array for each group were identified. For differential expression and multivariate analysis, background-corrected raw intensity data for the 67 samples were then loaded in to Perseus Software (*version 1.4.0.20*) followed by logarithmic normalization, data filtering and further analyses. Differentially expressed antigen between PCa, BPH and DC were identified (FDR = 0.01) using an independent sample *t*-test with Bonferroni correction for multiple testing. Further, unsupervised hierarchical clustering was performed to identify unique antigen signature for groups examined. Multivariate testing, using principal component analysis (PCA) was also performed to see how the groups clustered along each principal component. Hierarchical clustering was also performed using *k*-mean clustering on Cluster (*Version 3.0*) software in conjunction with Java TreeView (*version 3.0*) software. In addition to these analyses, the top 50 antigens were evaluated using a 3-way Venn diagram plotted using the software known as Venny (http://bioinfogp.cnb.csic.es/tools/venny/), a freely available online Venn diagram plotting resource. Antigens unique to PCa, BPH and DC were identified and compared with those identified by linear and differential expression analyses. Top 20 antigens with the highest signal intensities in PCa, BPH and DC were also analysed. Racial variation in PCa antigen expression was examined and high ranking antigen expression in Africans, Mixed ancestry and Caucasians in our PCa cohort were identified. We also confirmed the presence of potential antigen biomarkers of PCa in our previous shotgun urinary PCa proteomic data [45].

### Functional pathway analysis

Functional Pathway enrichment analyses of highly expressed antigen by linear expression, differential expression and Venn diagram analysis were performed using the GeneMANIA software (http://www.genemania.org/), a free online gene interaction pathway analysis tool. Functionally enriched genes and pathways were then confirmed using the STRING (*version 9.1*) functional protein association network software (freely available at http://string-db.org/).

## SUPPLEMENTARY MATERIAL FIGURES AND TABLES



## References

[1] Ferlay J, Soerjomataram I, Dikshit R, Eser S, Mathers C, Rebelo M, Parkin DM, Forman D, Bray F (2015). Cancer incidence and mortality worldwide: sources, methods and major patterns in GLOBOCAN 2012. International journal of cancer.

[2] Ross RK, Coetzee GA, Reichardt J, Skinner E, Henderson BE (1995). Does the Racial-Ethnic Variation in Prostate-Cancer Risk Have a Hormonal Basis. Cancer.

[3] Pettaway CA (1999). Racial differences in the androgen/androgen receptor pathway in prostate cancer. Journal of the National Medical Association.

[4] Freedman ML, Pearce CL, Penney KL, Hirschhorn JN, Kolonel LN, Henderson BE, Altshuler D (2005). Systematic evaluation of genetic variation at the androgen receptor locus and risk of prostate cancer in a multiethnic cohort study. American journal of human genetics.

[5] Sartor O, Zheng Q, Eastham JA (1999). Androgen receptor gene CAG repeat length varies in a race-specific fashion in men without prostate cancer. Urology.

[6] Hayes RB, Ziegler RG, Gridley G, Swanson C, Greenberg RS, Swanson GM, Schoenberg JB, Silverman DT, Brown LM, Pottern LM, Liff J, Schwartz AG, Fraumeni JF, Hoover RN (1999). Dietary factors and risks for prostate cancer among blacks and whites in the United States. Cancer epidemiology, biomarkers & prevention.

[7] Hollis BW, Marshall DT, Savage SJ, Garrett-Mayer E, Kindy MS, Gattoni-Celli S (2013). Vitamin D3 supplementation, low-risk prostate cancer, and health disparities. The Journal of steroid biochemistry and molecular biology.

[8] Powell IJ (2011). The precise role of ethnicity and family history on aggressive prostate cancer: a review analysis. [Article in English, Spanish]. Archivos espanoles de urologia.

[9] Martin DN, Starks AM, Ambs S (2013). Biological determinants of health disparities in prostate cancer. Current opinion in oncology.

[10] Powell IJ, Dyson G, Land S, Ruterbusch J, Bock CH, Lenk S, Herawi M, Everson R, Giroux CN, Schwartz AG, Bollig-Fischer A (2013). Genes associated with prostate cancer are differentially expressed in African American and European American men. Cancer epidemiology, biomarkers & prevention.

[11] Dias SJ, Zhou X, Ivanovic M, Gailey MP, Dhar S, Zhang L, He Z, Penman AD, Vijayakumar S, Levenson AS (2013). Nuclear MTA1 overexpression is associated with aggressive prostate cancer, recurrence and metastasis in African Americans. Scientific reports.

[12] Filella X, Foj L (2015). Emerging biomarkers in the detection and prognosis of prostate cancer. Clinical chemistry and laboratory medicine.

[13] Liong ML, Lim CR, Yang H, Chao S, Bong CW, Leong WS, Das PK, Loh CS, Lau BE, Yu CG, Ooi EJ, Nam RK, Allen PD, Steele GS, Wassmann K, Richie JP (2012). Blood-based biomarkers of aggressive prostate cancer. PloS one.

[14] Al-Ruwaili JA, Larkin SE, Zeidan BA, Taylor MG, Adra CN, Aukim-Hastie CL, Townsend PA (2010). Discovery of serum protein biomarkers for prostate cancer progression by proteomic analysis. Cancer genomics & proteomics.

[15] Velonas VM, Woo HH, dos Remedios CG, Assinder SJ (2013). Current status of biomarkers for prostate cancer. International journal of molecular sciences.

[16] Hernandez J, Thompson IM (2004). Prostate-specific antigen: a review of the validation of the most commonly used cancer biomarker. Cancer.

[17] Quintero IB, Araujo CL, Pulkka AE, Wirkkala RS, Herrala AM, Eskelinen EL, Jokitalo E, Hellstrom PA, Tuominen HJ, Hirvikoski PP, Vihko PT (2007). Prostatic acid phosphatase is not a prostate specific target. Cancer research.

[18] Graddis TJ, McMahan CJ, Tamman J, Page KJ, Trager JB (2011). Prostatic acid phosphatase expression in human tissues. International journal of clinical and experimental pathology.

[19] Stephan C, Ralla B, Jung K (2014). Prostate-specific antigen and other serum and urine markers in prostate cancer. Biochimica et biophysica acta.

[20] Bratt O, Lilja H (2015). Serum markers in prostate cancer detection. Current opinion in urology.

[21] Dijkstra S, Mulders PF, Schalken JA (2014). Clinical use of novel urine and blood based prostate cancer biomarkers: a review. Clinical biochemistry.

[22] Behesnilian AS, Reiter RE (2015). Risk stratification of prostate cancer in the modern era. Current opinion in urology.

[23] Zhang DY, Ye F, Gao L, Liu X, Zhao X, Che Y, Wang H, Wang L, Wu J, Song D, Liu W, Xu H, Jiang B, Zhang W, Wang J, Lee P (2009). Proteomics, pathway array and signaling network-based medicine in cancer. Cell division.

[24] Rho JH, Lampe PD (2013). High-throughput screening for native autoantigen-autoantibody complexes using antibody microarrays. Journal of proteome research.

[25] Gnjatic S, Ritter E, Buchler MW, Giese NA, Brors B, Frei C, Murray A, Halama N, Zornig I, Chen YT, Andrews C, Ritter G, Old LJ, Odunsi K, Jager D (2010). Seromic profiling of ovarian and pancreatic cancer. Proceedings of the National Academy of Sciences of the United States of America.

[26] Blackburn JM, Shoko A (2011). Protein function microarrays for customised systems-oriented proteome analysis. Methods in molecular biology.

[27] Liu BC, Dijohnson DA, O'Rourke DJ (2012). Antibody profiling with protein antigen microarrays in early stage cancer. Expert opinion on medical diagnostics.

[28] Alhamdani MS, Schroder C, Hoheisel JD (2009). Oncoproteomic profiling with antibody microarrays. Genome medicine.

[29] Haab BB (2003). Methods and applications of antibody microarrays in cancer research. Proteomics.

[30] Haab BB (2005). Antibody arrays in cancer research. Molecular & cellular proteomics.

[31] Cho-Chung YS (2006). Autoantibody biomarkers in the detection of cancer. Biochimica et biophysica acta.

[32] Stempfer R, Syed P, Vierlinger K, Pichler R, Meese E, Leidinger P, Ludwig N, Kriegner A, Nohammer C, Weinhausel A (2010). Tumour auto-antibody screening: performance of protein microarrays using SEREX derived antigens. BMC cancer.

[33] Simpson AJ, Caballero OL, Jungbluth A, Chen YT, Old LJ (2005). Cancer/testis antigens, gametogenesis and cancer. Nature reviews Cancer.

[34] Kalejs M, Erenpreisa J (2005). Cancer/testis antigens and gametogenesis: a review and “brain-storming” session. Cancer cell international.

[35] Balafoutas D, zur Hausen A, Mayer S, Hirschfeld M, Jaeger M, Denschlag D, Gitsch G, Jungbluth A, Stickeler E (2013). Cancer testis antigens and NY-BR-1 expression in primary breast cancer: prognostic and therapeutic implications. BMC cancer.

[36] Beeton-Kempen N, Duarte J, Shoko A, Serufuri JM, John T, Cebon J, Blackburn J (2014). Development of a novel, quantitative protein microarray platform for the multiplexed serological analysis of autoantibodies to cancer-testis antigens. International journal of cancer.

[37] Fratta E, Coral S, Covre A, Parisi G, Colizzi F, Danielli R, Nicolay HJ, Sigalotti L, Maio M (2011). The biology of cancer testis antigens: putative function, regulation and therapeutic potential. Molecular oncology.

[38] Kulkarni P, Shiraishi T, Rajagopalan K, Kim R, Mooney SM, Getzenberg RH (2012). Cancer/testis antigens and urological malignancies. Nat Rev Urol.

[39] Gnjatic S, Wheeler C, Ebner M, Ritter E, Murray A, Altorki NK, Ferrara CA, Hepburne-Scott H, Joyce S, Koopman J, McAndrew MB, Workman N, Ritter G, Fallon R, Old LJ (2009). Seromic analysis of antibody responses in non-small cell lung cancer patients and healthy donors using conformational protein arrays. Journal of immunological methods.

[40] Takahashi S, Shiraishi T, Miles N, Trock BJ, Kulkarni P, Getzenberg RH (2015). Nanowire analysis of cancer-testis antigens as biomarkers of aggressive prostate cancer. Urology.

[41] Shiraishi T, Getzenberg RH, Kulkarni P (2012). Cancer/testis antigens: novel tools for discerning aggressive and non-aggressive prostate cancer. Asian journal of andrology.

[42] Suyama T, Shiraishi T, Zeng Y, Yu W, Parekh N, Vessella RL, Luo J, Getzenberg RH, Kulkarni P (2010). Expression of cancer/testis antigens in prostate cancer is associated with disease progression. The Prostate.

[43] Cheema Z, Hari-Gupta Y, Kita GX, Farrar D, Seddon I, Corr J, Klenova E (2014). Expression of the cancer-testis antigen BORIS correlates with prostate cancer. The Prostate.

[44] Shiraishi T, Terada N, Zeng Y, Suyama T, Luo J, Trock B, Kulkarni P, Getzenberg RH (2011). Cancer/Testis Antigens as potential predictors of biochemical recurrence of prostate cancer following radical prostatectomy. Journal of translational medicine.

[45] Adeola HA, Soares NC, Paccez JD, Kaestner L, Blackburn JM, Zerbini LF (2015). Discovery of novel candidate urinary protein biomarkers for prostate cancer in a multiethnic cohort of South African patients *via* label-free mass spectrometry. Proteomics Clinical applications.

[46] Kingsmore SF (2006). Multiplexed protein measurement: technologies and applications of protein and antibody arrays. Nature reviews Drug discovery.

[47] Burbelo PD, Ching KH, Bush ER, Han BL, Iadarola MJ (2010). Antibody-profiling technologies for studying humoral responses to infectious agents. Expert review of vaccines.

[48] Tabakman SM, Lau L, Robinson JT, Price J, Sherlock SP, Wang H, Zhang B, Chen Z, Tangsombatvisit S, Jarrell JA, Utz PJ, Dai H (2011). Plasmonic substrates for multiplexed protein microarrays with femtomolar sensitivity and broad dynamic range. Nature communications.

[49] Dudas SP, Chatterjee M, Tainsky MA (2010). Usage of cancer associated autoantibodies in the detection of disease. Cancer biomarkers : section A of Disease markers.

[50] Chatterjee M, Draghici S, Tainsky MA (2006). Immunotheranostics: breaking tolerance in immunotherapy using tumor autoantigens identified on protein microarrays. Current opinion in drug discovery & development.

[51] Tojo A, Kinugasa S (2012). Mechanisms of glomerular albumin filtration and tubular reabsorption. International journal of nephrology.

[52] Couturier MR, Graf EH, Griffin AT (2014). Urine antigen tests for the diagnosis of respiratory infections: legionellosis, histoplasmosis, pneumococcal pneumonia. Clinics in laboratory medicine.

[53] Sinclair A, Xie X, Teltscher M, Dendukuri N (2013). Systematic review and meta-analysis of a urine-based pneumococcal antigen test for diagnosis of community-acquired pneumonia caused by Streptococcus pneumoniae. Journal of clinical microbiology.

[54] Choudhry V, Saxena RK (2002). Detection of Mycobacterium tuberculosis antigens in urinary proteins of tuberculosis patients. European journal of clinical microbiology & infectious diseases.

[55] Young BL, Mlamla Z, Gqamana PP, Smit S, Roberts T, Peter J, Theron G, Govender U, Dheda K, Blackburn J (2014). The identification of tuberculosis biomarkers in human urine samples. The European respiratory journal.

[56] Holmquist P, Sjoblad S, Torffvit O (2004). Pore size and charge selectivity of the glomerular membrane at the time of diagnosis of diabetes. Pediatr Nephrol.

[57] Haraldsson B, Nystrom J, Deen WM (2008). Properties of the glomerular barrier and mechanisms of proteinuria. Physiological reviews.

[58] Kurts C, Panzer U, Anders HJ, Rees AJ (2013). The immune system and kidney disease: basic concepts and clinical implications. Nature reviews Immunology.

[59] Gottschalk C, Damuzzo V, Gotot J, Kroczek RA, Yagita H, Murphy KM, Knolle PA, Ludwig-Portugall I, Kurts C (2013). Batf3-dependent dendritic cells in the renal lymph node induce tolerance against circulating antigens. Journal of the American Society of Nephrology.

[60] Pindjakova J, Griffin MD (2013). The renal lymph node and immune tolerance to filtered antigens. Journal of the American Society of Nephrology.

[61] Mishra DK, Chen Z, Wu Y, Sarkissyan M, Koeffler HP, Vadgama JV (2010). Global methylation pattern of genes in androgen-sensitive and androgen-independent prostate cancer cells. Molecular cancer therapeutics.

[62] Atanackovic D, Luetkens T, Kloth B, Fuchs G, Cao Y, Hildebrandt Y, Meyer S, Bartels K, Reinhard H, Lajmi N, Hegewisch-Becker S, Schilling G, Platzbecker U, Kobbe G, Schroeder T, Bokemeyer C (2011). Cancer-testis antigen expression and its epigenetic modulation in acute myeloid leukemia. American journal of hematology.

[63] Kouprina N, Noskov VN, Solomon G, Otstot J, Isaacs W, Xu J, Schleutker J, Larionov V (2007). Mutational analysis of SPANX genes in families with X-linked prostate cancer. The Prostate.

[64] Yao S, Ireland SJ, Bee A, Beesley C, Forootan SS, Dodson A, Dickinson T, Gerard P, Lian LY, Risk JM, Smith P, Malki MI, Ke Y, Cooper CS, Gosden C, Foster CS (2012). Splice variant PRKC-zeta(−PrC) is a novel biomarker of human prostate cancer. British journal of cancer.

[65] Zabransky DJ, Smith HA, Thoburn CJ, Zahurak M, Keizman D, Carducci M, Eisenberger MA, McNeel DG, Drake CG, Antonarakis ES (2012). Lenalidomide modulates IL-8 and anti-prostate antibody levels in men with biochemically recurrent prostate cancer. The Prostate.

[66] Hakem A, Bohgaki M, Lemmers B, Tai E, Salmena L, Matysiak-Zablocki E, Jung YS, Karaskova J, Kaustov L, Duan S, Madore J, Boutros P, Sheng Y, Chesi M, Bergsagel PL, Perez-Ordonez B (2011). Role of Pirh2 in mediating the regulation of p53 and c-Myc. PLoS genetics.

[67] Dai C, Gu W (2010). p53 post-translational modification: deregulated in tumorigenesis. Trends in molecular medicine.

[68] Lee SJ, Lee SH, Yoon MH, Park BJ (2013). A new p53 target gene, RKIP, is essential for DNA damage-induced cellular senescence and suppression of ERK activation. Neoplasia.

[69] Suppiah A, Greenman J (2013). Clinical utility of anti-p53 auto-antibody: systematic review and focus on colorectal cancer. World journal of gastroenterology.

[70] Amini S, Fathi F, Mobalegi J, Sofimajidpour H, Ghadimi T (2014). The expressions of stem cell markers: Oct4, Nanog, Sox2, nucleostemin, Bmi, Zfx, Tcl1, Tbx3, Dppa4, and Esrrb in bladder, colon, and prostate cancer, and certain cancer cell lines. Anatomy & cell biology.

[71] Duan Z, Duan Y, Lamendola DE, Yusuf RZ, Naeem R, Penson RT, Seiden MV (2003). Overexpression of MAGE/GAGE genes in paclitaxel/doxorubicin-resistant human cancer cell lines. Clinical cancer research.

[72] Wang H, Zhou W, Zheng Z, Zhang P, Tu B, He Q, Zhu WG (2012). The HDAC inhibitor depsipeptide transactivates the p53/p21 pathway by inducing DNA damage. DNA repair.

[73] Chun HK, Chung KS, Kim HC, Kang JE, Kang MA, Kim JT, Choi EH, Jung KE, Kim MH, Song EY, Kim SY, Won M, Lee HG (2010). OIP5 is a highly expressed potential therapeutic target for colorectal and gastric cancers. BMB reports.

[74] Zhang JY (2007). Mini-array of multiple tumor-associated antigens to enhance autoantibody detection for immunodiagnosis of hepatocellular carcinoma. Autoimmunity reviews.

[75] Li L, Wang K, Dai L, Wang P, Peng XX, Zhang JY (2008). Detection of autoantibodies to multiple tumor-associated antigens in the immunodiagnosis of ovarian cancer. Molecular medicine reports.

[76] Ye H, Sun C, Ren P, Dai L, Peng B, Wang K, Qian W, Zhang J (2013). Mini-array of multiple tumor-associated antigens (TAAs) in the immunodiagnosis of breast cancer. Oncology letters.

[77] Bunger S, Haug U, Kelly M, Posorski N, Klempt-Giessing K, Cartwright A, Fitzgerald SP, Toner V, McAleer D, Gemoll T, Laubert T, Buning J, Fellermann K, Bruch HP, Roblick UJ, Brenner H (2012). A novel multiplex-protein array for serum diagnostics of colon cancer: a case-control study. BMC cancer.

[78] de Martel C, Ferlay J, Franceschi S, Vignat J, Bray F, Forman D, Plummer M (2012). Global burden of cancers attributable to infections in 2008: a review and synthetic analysis. The lancet oncology.

[79] Viljoen KS, Dakshinamurthy A, Goldberg P, Blackburn JM (2015). Quantitative profiling of colorectal cancer-associated bacteria reveals associations between fusobacterium spp., enterotoxigenic Bacteroides fragilis (ETBF) and clinicopathological features of colorectal cancer. PloS one.

[80] Kyi C, Postow MA (2014). Checkpoint blocking antibodies in cancer immunotherapy. FEBS letters.

[81] Park TS, Rosenberg SA, Morgan RA (2011). Treating cancer with genetically engineered T cells. Trends in biotechnology.

[82] Joniau S, Abrahamsson PA, Bellmunt J, Figdor C, Hamdy F, Verhagen P, Vogelzang NJ, Wirth M, Van Poppel H, Osanto S (2012). Current vaccination strategies for prostate cancer. European urology.

[83] Baxevanis CN, Papamichail M, Perez SA (2015). Prostate cancer vaccines: the long road to clinical application. Cancer immunology, immunotherapy.

[84] Duarte J, Serufuri J., Mulder N., Blackburn J, Wang X (2013). Protein Function Microarrays: Design, Use and Bioinformatic Analysis in Cancer Biomarker Discovery and Quantitation. Bioinformatics of Human Proteomics.

