# Supplementary material for: Novel potential serological prostate cancer biomarkers using CT100+ cancer antigen microarray platform in a multi-cultural South African cohort
Source: Oncotarget. 2016 Feb 12;7(12):13945–64. doi: 10.18632/oncotarget.7359 (PMC4924690; doi:10.18632/oncotarget.7359)
Supplement: Supplementary file 1 [file oncotarget-07-13945-s001.pdf]

Novel potential serological prostate cancer biomarkers using CT100+ cancer antigen microarray platform in a multi-cultural South African cohort

Supplementary Material

Supplementary Figure 1. Annotation of antigens printed on the array

BAGE2, BAGE3, BAGE4, BAGE5, CCDC33, CEP290, COL6A1, COX6B2, CSAG2, CT47.11, CT62, CTAG2, CXorf48.1, DDX53, DSCR8/MMA1, FTHL17, GAGE1, GAGE2A, GAGE4, GAGE5, GAGE6, GAGE7, GRWD1, HORMAD1, LDHC, LEMD1, LIP1, MAGEA1, MAGEA10, MAGEA11, MAGEA2, MAGEA3, MAGEA4v2, MAGEA4v3, MAGEA4v4, MAGEA5, MAGEB1, MAGEB5, MAGEB6, MART-1, MICA, NLRP4, NXF2, NY-CO-45, NY-ESO-1, OIP5, p53, PBK, RELT, ROPN1, SGY-1, SILV, SPAG9, SPANXA1, SPANXB1, SPANXC, SPANXD, SPO11, SSX1, SSX2A, SSX4, SYCE1, SYCP1, THEG, TPTE, TSGA10, TSSK6, TYR, XAGE-2, XAGE3av1, XAGE3av2, ZNF165, AKT1, CDK2, CDK4, CDK7, FES, FGFR2, MAPK1, MAPK3, PRKCZ, RAF, SRC, CALM1, CDC25A, CREB1, CTNNB1, p53 S6A, p53 C141Y, p53 S15A, P53 T18A, p53 Q136x, p53 S46A, p53 K382R, p53 S392A, p53 M133T, p53 L344P, cytochrome P450 3A4, cytochrome P450 reductase, EGFR, 5T4/TPBG, XAGE1B, SOX2, ACVR2A, ACVR2B, ITGB1, MAP9, PIM1, TKTL1 (isoform a), SPATS1 (isoform a), DPPA2, SOX1, ROPN1A, CEACAM 1 isoform 1, POU5F1/OCT3/OCT4A var. 1, NANOG, BORIS B0, DPPA4, DPPA3, GDF3, CTAG2/LAGE-1b/LAGE-1L, CAMEL and NY-ESO-1 ORF2

Supplementary Figure 2. TIFF image variation in antigen intensities

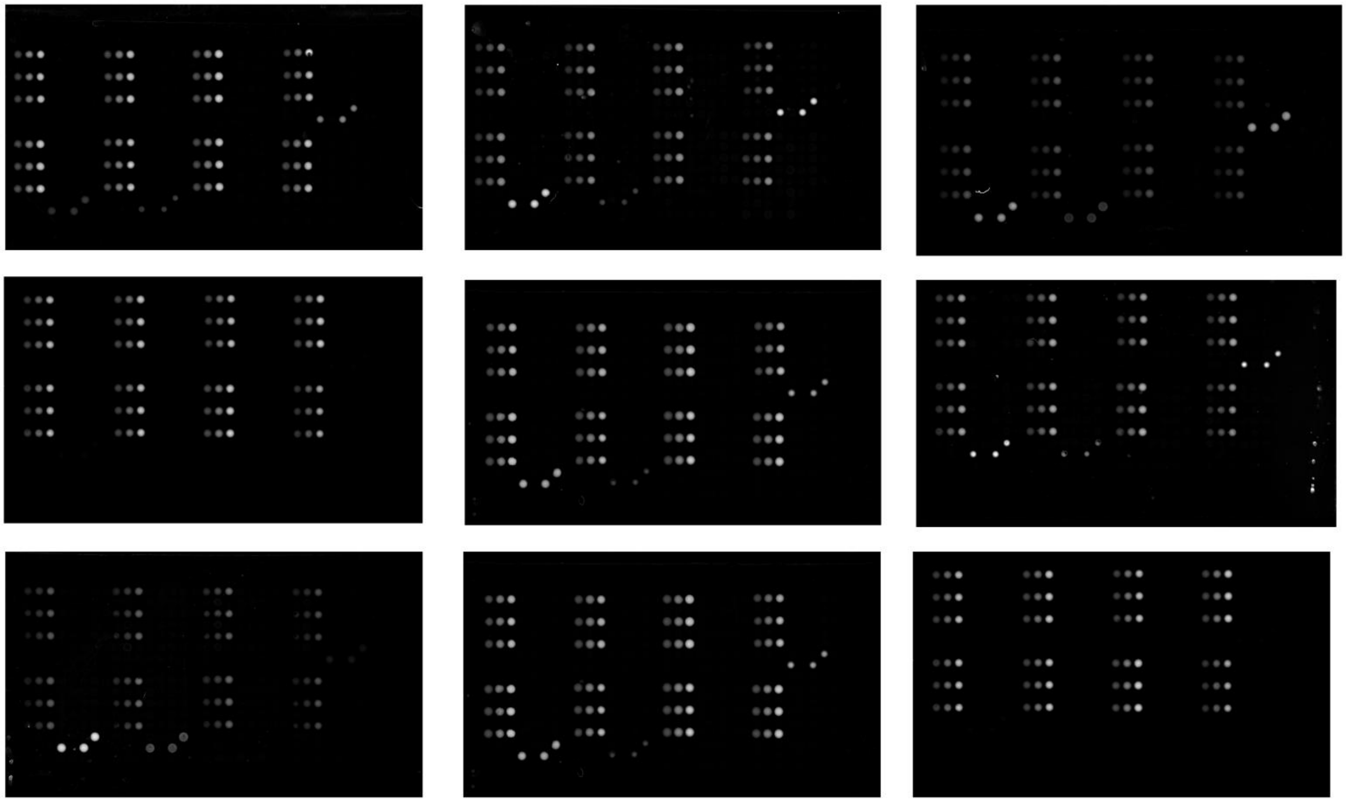

Supplementary Table 1. Top 20 Antigens with highest mean intensities

PCa		BPH		DC	
Antigens	Mean Intensities	Antigens	Mean Intensities	Antigens	Mean Intensity
PRKCZ	1048.269	PRKCZ	906.9973	DDX53	813.5409
p53 S15A	938.7786	MAGEB1	846.6051	MAGEB1	778.1442
OIP5	849.06	GAGE2A	778.3152	PRKCZ	757.3264
MAPK3	846.1652	GAGE1	766.9259	GAGE4	668.5862
MAGEB1	820.2938	CCDC33	764.3024	GAGE1	650.7049
NY-ESO-1	816.6235	GAGE5	734.0895	GAGE6	648.4141
ROPN1A	810.1449	LEMD1	715.8789	p53 S15A	616.0384
p53 C141Y	702.5416	OIP5	685.995	OIP5	600.2059
P53 T18A	692.4752	CTNNB1	625.9252	MAPK3	598.9191
p53 S46A	691.9831	GAGE7	612.5261	MAGEB5	584.3317
LDHC	690.7583	RAF	604.4013	MAGEA2	583.7003
CDC25A	683.7474	MAGEB5	591.5896	PBK	570.5075
CTNNB1	678.2069	MAGEA10	569.3561	p53 Q136x	568.274
CAMEL	665.2231	GAGE6	558.6041	CALM1	565.0479
GAGE1	653.8799	CDC25A	555.083	LEMD1	560.5245
p53 S6A	632.2697	p53 S15A	551.1414	MAGEB6	551.0759
XAGE3av1	632.0129	p53 C141Y	535.9077	MAGEA10	545.1686
GAGE6	629.6645	CREB1	526.4242	CT47.11	540.9317
FES	607.1422	GAGE4	500.2994	P53 T18A	532.3706
MAGEB5	600.0561	MAGEA2	496.1574	NY-ESO-1	529.8623

Supplementary table 2. Venn diagram analysis result

Unique to PCa	Unique to BPH	Unique to DC
CDK7	CSAG2	DDX53
SPANXA1	SSX4	p53 Q136x
p53 S392A	SPATS1 (isoform a)	p53
DPPA4	BAGE2	CALM1
LDHC	CDK4	CT47.11
FGFR2	ACVR2A	NY-ESO-1 ORF2
BORIS B0	XAGE3av2	DPPA2
TPTE	GAGE7	DPPA3
DSCR8/MMA1	AKT1	HORMAD1
ACVR2B	CTAG2	MART-1
SPO11		COL6A1
THEG		cytochrome P450 reductase
		SRC
		TSSK6
		CTAG2/LAGE-1b/LAGE-1L

Some potential PCa antigen biomarkers by Venn diagram analysis result for PCa, BPH and DC shows overlap with those from linear and differential analyses.

Supplementary table 3. Differentially expressed antigens by independent sample t-test

A. PCa AND BPH

Upregulated	Downregulated
1. DPPA4	1. GAGE5
2. CECAM1 Isoform 1	2. MAGEB5
3. NY-ESO-1	3. EGFR
4. P53 L344P	4. CCDC33
	5. CSAG2

B. PCa AND DC

Upregulated	Downregulated
1.P53 L344P	1.GAGE5
2.RAF	2.DDX53
3.DPPA4	3.CT47.11
4.ZNF165	4.P53
5.TKTL1 (Isoform a)	5.P53 Q136X

C. PCa AND CONTROLS

Upregulated	Downregulated
1.DPPA4	1.P53 Q136X
2.P53 L344P	2.MAGEB6
3.MAPK3	3.MAGEB5
4.CAMEL	4.GAGE5
5.RAF	5.CSAG2
6.TKTL1 (Isoform a)	6.PBK
7.LDHC	
8.P53 C141Y	
9.NY-ESO-1	
10.P53 K328R	
11.P53 S15A	
12.CDK2	
13.MAGE11	
14.FES	
15.P53 T18A	
16.OIP5	
17.SSX2A	

Differentially expressed antigens by independent sample t-test comparing **(A)** PCa to BPH; **(B)** PCa to DC; and **(C)** PCa to all controls (DC & BPH).

Supplementary Table 4. Functional annotation of antigen

A. Differentially expressed between PCa and DC

Function	FDR	Coverage
1 query genes	n/a	11 / 11
2 Fc receptor signaling pathway	2.74E-7	9 / 219
3 epidermal growth factor receptor signaling pathway	2.74E-7	9 / 213
4 ERBB signaling pathway	2.74E-7	9 / 216
5 Fc-epsilon receptor signaling pathway	7.81E-7	8 / 166
6 neurotrophin signaling pathway	7.81E-7	9 / 277
7 neurotrophin TRK receptor signaling pathway	7.81E-7	9 / 274
8 cellular response to fibroblast growth factor stimulus	7.81E-7	8 / 183
9 fibroblast growth factor receptor signaling pathway	7.81E-7	8 / 170
10 response to fibroblast growth factor	7.81E-7	8 / 183
11 immune response-regulating cell surface receptor signaling pathway	1.23E-6	9 / 297
12 ERK1 and ERK2 cascade	5.76E-5	6 / 120
13 protein autophosphorylation	1.82E-4	6 / 149
14 positive regulation of MAPK cascade	1.82E-4	7 / 253
15 response to steroid hormone	2.46E-4	5 / 81
16 positive regulation of ERK1 and ERK2 cascade	2.6E-4	5 / 83
17 blood vessel morphogenesis	3.15E-4	6 / 170
18 regulation of ion homeostasis	5.83E-4	5 / 100
19 blood vessel development	5.9E-4	6 / 193
20 cell-matrix adhesion	6.04E-4	5 / 103
21 regulation of ERK1 and ERK2 cascade	8.33E-4	5 / 111
22 response to estrogen	8.48E-4	4 / 47
23 skin morphogenesis	9.29E-4	3 / 13

B. Differentially expressed between PCa and all controls (BPH and DC)

Function	FDR	Coverage
1 query genes	n/a	19 / 19
2 Fc-epsilon receptor signaling pathway	3.74E-4	7 / 166
3 Ras protein signal transduction	3.74E-4	7 / 167
4 Fc receptor signaling pathway	1.59E-3	7 / 219
5 fibroblast growth factor receptor signaling pathway	2.83E-3	6 / 170
6 neurotrophin TRK receptor signaling pathway	2.83E-3	7 / 274
7 immune response-regulating cell surface receptor signaling pathway	2.83E-3	7 / 297
8 cellular response to fibroblast growth factor stimulus	2.83E-3	6 / 183
9 neurotrophin signaling pathway	2.83E-3	7 / 277
10 response to insulin	2.83E-3	6 / 186
11 response to fibroblast growth factor	2.83E-3	6 / 183
12 cellular response to insulin stimulus	2.83E-3	6 / 182
13 small GTPase mediated signal transduction	2.83E-3	7 / 291
14 insulin receptor signaling pathway	2.83E-3	6 / 153
15 epidermal growth factor receptor signaling pathway	5.27E-3	6 / 213
16 ERBB signaling pathway	5.27E-3	6 / 216
17 ERK1 and ERK2 cascade	5.27E-3	5 / 120
18 cellular response to peptide hormone stimulus	9.98E-3	6 / 244
19 cellular response to peptide	1.01E-2	6 / 247
20 response to peptide hormone	1.15E-2	6 / 255
21 response to peptide	1.22E-2	6 / 260
22 positive regulation of ERK1 and ERK2 cascade	2.14E-2	4 / 83
23 cellular process involved in reproduction	5.06E-2	5 / 207
24 regulation of ERK1 and ERK2 cascade	6.1E-2	4 / 111
25 activation of MAPKK activity	7.66E-2	3 / 44

C. Differentially expressed by Venn diagram analysis

Function	FDR	Coverage
1 query genes	n/a	12 / 12
2 synapsis	3.2E-2	3 / 17
3 chromosome organization involved in meiosis	3.2E-2	3 / 18
4 cellular process involved in reproduction	1.38E-1	5 / 207
5 meiosis I	3.71E-1	3 / 50
6 synaptonemal complex assembly	3.82E-1	2 / 10
7 synaptonemal complex organization	3.82E-1	2 / 10
8 meiotic nuclear division	8.59E-1	3 / 84
9 meiotic cell cycle	8.59E-1	3 / 87
10 synaptonemal complex	8.59E-1	2 / 18

D. Protein interaction annotation of antigens with STRING 9.1

Term	# of Genes	p-value	p-value_fdr	p-value_bonferroni
fibroblast growth factor receptor signaling pathway	8	1.68E-07	2.12E-03	2.12E-03
cellular response to fibroblast growth factor stimulus	8	4.64E-07	2.33E-03	5.85E-03
response to fibroblast growth factor	8	5.54E-07	2.33E-03	6.98E-03
epidermal growth factor receptor signaling pathway	8	1.17E-06	2.67E-03	1.48E-02
ERBB signaling pathway	8	1.27E-06	2.67E-03	1.60E-02
reproduction	17	1.78E-06	2.67E-03	2.24E-02
neurotrophin TRK receptor signaling pathway	9	1.79E-06	2.67E-03	2.25E-02
neurotrophin signaling pathway	9	1.84E-06	2.67E-03	2.32E-02
cell division	12	2.19E-06	2.67E-03	2.76E-02
cell cycle	17	2.30E-06	2.67E-03	2.91E-02
Fc receptor signaling pathway	9	2.33E-06	2.67E-03	2.94E-02
single organism reproductive process	15	3.63E-06	3.02E-03	4.57E-02
response to epidermal growth factor	4	3.64E-06	3.02E-03	4.59E-02
positive regulation of cyclin-dependent protein serine/threonine kinase activity involved in G1/S transition of mitotic cell cycle	3	3.64E-06	3.02E-03	4.59E-02
positive regulation of phosphorylation	13	3.73E-06	3.02E-03	4.71E-02
Fc-epsilon receptor signaling pathway	8	3.84E-06	3.02E-03	4.84E-02
response to oxygen-containing compound	16	4.86E-06	3.60E-03	6.12E-02
regulation of cyclin-dependent protein serine/threonine kinase activity involved in G1/S transition of mitotic cell cycle	3	5.44E-06	3.80E-03	6.86E-02
regulation of cell fate commitment	4	5.73E-06	3.80E-03	7.22E-02
synapsis	4	6.59E-06	4.02E-03	8.30E-02
cellular process involved in reproduction	10	6.69E-06	4.02E-03	8.44E-02
cell fate specification	5	7.48E-06	4.07E-03	9.43E-02
cellular response to mechanical stimulus	5	7.48E-06	4.07E-03	9.43E-02
positive regulation of cell cycle	6	7.75E-06	4.07E-03	9.77E-02
positive regulation of MAPK cascade	9	8.34E-06	4.21E-03	1.05E-01
regulation of ERK1 and ERK2 cascade	5	2.84E-04	2.92E-02	1.00E+00

Functional pathway annotation of differentially expressed antigens by biologic processes shows remarkable overlap between top ranking processes by **(A)** differentially expressed antigens between PCa

and DC; **(B)** differentially expressed antigens between PCa and all controls; **(C)** antigens unique to PCa by Venn diagram analysis; and **(D)** interaction between all 41 potential biomarker antigens.

Supplementary table 5. Shotgun urinary proteomics verification of cancer antigens

Antigen	Homologous genes detected	Presence in cancer antigen microarray data			Presence in Shotgun data	Presence in PCa shotgun	Biomarker Category
		BP	DC	PC			
FGFR2	FGFR2	low	low	high	yes	yes	Moderate
MAPK1	yes	low	low	high	yes	no	weak
COL6A1	COL6A1	low	low	very high	yes	yes	Strong
SOX1	yes	low	low	high	yes	no	Weak
CALM1	CALM1	low	low	high	yes	yes	Moderate
SRC	yes	low	high	low	yes	no	Weak
LIP1	yes	high	high	high	yes	no	Weak
CEACAM 1 isoform 1	yes	low	high	low	yes	no	Weak
RELT	RELT	low	high	high	yes	no	Weak
EGFR	yes	medium	high	low	yes	no	weak
ITGB1	ITGB1	high	low	low	yes	no	Weak

BP= Benign prostatic hyperplasia; **DC**= Disease control; **PC**= Prostate cancer

Shotgun urinary proteomics verification of cancer antigens show 11 cancer antigens on the CT100+ were also present in urinary shotgun proteomics result. Only FGFR2, COL6A1 and CALM1 were found to be strongly expressed in PCa in both database, hence are potentially useful antigen biomarkers of PCa.
